# Baseline Inflammatory Status Reveals Dichotomic Immune Mechanisms Involved In Primary-Progressive Multiple Sclerosis Pathology

**DOI:** 10.3389/fimmu.2022.842354

**Published:** 2022-03-21

**Authors:** José I. Fernández-Velasco, Enric Monreal, Jens Kuhle, Virginia Meca-Lallana, José Meca-Lallana, Guillermo Izquierdo, Celia Oreja-Guevara, Francisco Gascón-Giménez, Susana Sainz de la Maza, Paulette E. Walo-Delgado, Paloma Lapuente-Suanzes, Aleksandra Maceski, Eulalia Rodríguez-Martín, Ernesto Roldán, Noelia Villarrubia, Albert Saiz, Yolanda Blanco, Carolina Diaz-Pérez, Gabriel Valero-López, Judit Diaz-Diaz, Yolanda Aladro, Luis Brieva, Cristina Íñiguez, Inés González-Suárez, Luis A Rodríguez de Antonio, José M. García-Domínguez, Julia Sabin, Sara Llufriu, Jaime Masjuan, Lucienne Costa-Frossard, Luisa M. Villar

**Affiliations:** ^1^Immunology Department, Ramon y Cajal University Hospital, Madrid, Spain; ^2^Neurology Department, Ramon y Cajal University Hospital, Madrid, Spain; ^3^Neurologic Clinic and Policlinic, Departments of Medicine, Biomedicine, and Clinical Research, University Hospital Basel, University of Basel, Basel, Switzerland; ^4^Neurology Department, La Princesa University Hospital, Madrid, Spain; ^5^Multiple Sclerosis and Clinical Neuroimmunology Unit, Virgen de la Arrixaca University Hospital, Murcia, Spain; ^6^Multiple Sclerosis Unit, Vithas Nisa Sevilla Hospital, Sevilla, Spain; ^7^Neurology Department, Cliínico San Carlos Hospital, Instituto de Investigación Sanitaria San Carlos (IdISSC), Madrid, Spain; ^8^Neurology Department, Valencia Clinic University Hospital, Valencia, Spain; ^9^Center of Neuroimmunology, Neurology Department, Clínic of Barcelona Hospital, Institut d’Investigacions Biomèdiques August Pi i Sunyer (IDIBAPS), and Institut de Neurociències, Universitat de Barcelona, Barcelona, Spain; ^10^Neurology Department, Getafe University Hospital, Madrid, Spain; ^11^Neurology Department, Arnau de Vilanova Hospital, Lleida, Spain; ^12^Neurology Department, Lozano Blesa Clinic University Hospital, Zaragoza, Spain; ^13^Neurology Department, Alvaro Cunqueiro Hospital, Vigo, Spain; ^14^Neurology Department, Fuenlabrada University Hospital, Madrid, Spain; ^15^Neurology Department, Gregorio Marañón University Hospital, Madrid, Spain; ^16^Neurology Department, Puerta de Hierro University Hospital, Madrid, Spain

**Keywords:** multiple sclerosis, demyelinating diseases, ocrelizumab, B cells, biomarkers

## Abstract

**Objective:**

To ascertain the role of inflammation in the response to ocrelizumab in primary-progressive multiple sclerosis (PPMS).

**Methods:**

Multicenter prospective study including 69 patients with PPMS who initiated ocrelizumab treatment, classified according to baseline presence [Gd+, n=16] or absence [Gd-, n=53] of gadolinium-enhancing lesions in brain MRI. Ten Gd+ (62.5%) and 41 Gd- patients (77.4%) showed non-evidence of disease activity (NEDA) defined as no disability progression or new MRI lesions after 1 year of treatment. Blood immune cell subsets were characterized by flow cytometry, serum immunoglobulins by nephelometry, and serum neurofilament light-chains (sNfL) by SIMOA. Statistical analyses were corrected with the Bonferroni formula.

**Results:**

More than 60% of patients reached NEDA after a year of treatment, regardless of their baseline characteristics. In Gd+ patients, it associated with a low repopulation rate of inflammatory B cells accompanied by a reduction of sNfL values 6 months after their first ocrelizumab dose. Patients in Gd- group also had low B cell numbers and sNfL values 6 months after initiating treatment, independent of their treatment response. In these patients, NEDA status was associated with a tolerogenic remodeling of the T and innate immune cell compartments, and with a clear increase of serum IgA levels.

**Conclusion:**

Baseline inflammation influences which immunological pathways predominate in patients with PPMS. Inflammatory B cells played a pivotal role in the Gd+ group and inflammatory T and innate immune cells in Gd- patients. B cell depletion can modulate both mechanisms.

## 1 Introduction

Multiple sclerosis (MS) is the most common demyelinating disease of the central nervous system (1). It induces demyelination, inflammation, and axonal damage, responsible for the permanent neurological deficits suffered by patients with MS (2). Primary progressive MS (PPMS) represents about 10-15% (3) of all MS cases and is characterized by a disease progression that remains continuous since disease onset (4), with or without concomitant visible inflammation by conventional MRI. Although many therapeutic options are available for relapsing remitting MS (RRMS), this is not the case for PPMS. Most of the anti-inflammatory drugs found useful for patients with RRMS are not effective among those with PPMS (5). However, the anti CD20 antibody rituximab showed efficacy in depleting cerebrospinal fluid and peripheral blood B cells in PPMS (6) and results of the OLYMPUS clinical trial suggested that B cell depletion could be effective in those PPMS patients exhibiting signs of inflammation as demonstrated by the occurrence of contrast-enhancing lesions at baseline on MRI (7). Recently, results of the ORATORIO clinical trial with ocrelizumab, a humanized anti CD20 antibody, showed that patients with PPMS responded to this drug, independent of the presence of clinically demonstrable inflammation (8), and it was approved for the treatment of PPMS patients. Ocrelizumab not only induces B cell depletion but modulates T cell compartment toward adopting a more tolerogenic status (9).

Therefore, we aimed to explore the mechanisms associated with favorable response to ocrelizumab in inflammatory and non-inflammatory PPMS cases, toward facilitating the early identification of ocrelizumab responders and revealing new putative therapeutic targets in PPMS.

## 2 Materials and Methods

### 2.1 Patients

This multicenter, prospective longitudinal study included 69 patients with PPMS diagnosed according to the McDonald criteria (10) who consecutively initiated ocrelizumab treatment in 13 Spanish University Hospitals. Patients were subdivided into four groups based on their inflammatory status (presence [Gd+, n=16] or absence [Gd-, n=53] of gadolinium-enhancing lesions at baseline) and their response to 1 year of ocrelizumab treatment. Non evidence of disease activity (NEDA) was defined as the absence of further Expanded Disability-Status Scale (EDSS) progression with no new MRI lesions at 1 year; in contrast, evidence of disease activity (EDA) patients as having at least one of the above-mentioned conditions. We considered patients as having an increase in the EDSS score when this was confirmed three months after the first assessment. The maximum gap between baseline MRI and treatment initiation was a month.

### 2.2 Sample Collection

Blood samples were collected in heparinized tubes immediately before (baseline) and 6 months after (before the second dose) ocrelizumab treatment. In both cases they were obtained the day Ocrelizumab was administered, just before initiating the infusion. Samples were then sent to the Immunology Department at Ramón y Cajal University Hospital (Madrid). Peripheral blood mononuclear cells (PBMCs) were isolated from 20 mL of heparinized whole blood as previously described (9) and cryopreserved in fetal bovine serum (HyClone Laboratories) supplemented with 10% DMSO (dimethyl sulfoxide) until further analysis. The samples collected at baseline and 6 months were analyzed simultaneously to avoid inter-assay variability. Serum samples were also collected and stored at -80°C while awaiting analysis. Total lymphocyte and monocyte counts were determined using a Coulter Counter from 10 mL of fresh EDTA-treated blood.

### 2.3 Monoclonal Antibodies

**Figure 1:**
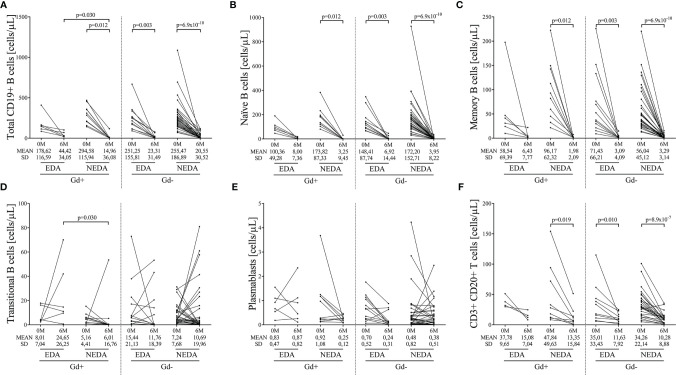
Changes in blood B cell subsets induced by ocrelizumab treatment. Total and B-cell subsets were obtained before (0M) and at 6 months (6M) of ocrelizumab treatment and classified based on their inflammatory status (presence [Gd+] or absence [Gd-] of gadolinium enhancing lesions at baseline) and response (optimal NEDA or suboptimal EDA) to treatment at one year of follow-up. N was 6, 10, 12 and 41 in Gd+ EDA, Gd+ NEDA, Gd- EDA and Gd- NEDA, respectively. Graphs showing changes in absolute numbers (cells/μL) of total CD19+ B cells **(A)**, naïve B cells **(B)**, memory B cells **(C)**, transitional B cells **(D)**, plasmablasts **(E)** and CD20 T cells **(F)**. SD, Standard deviation. Bonferroni-corrected p-values are shown.

The monoclonal antibodies used in this study are listed in **Supplementary Table 1**. No differences were observed in plasmablast counts in any of the groups studied (**Figure 1E**; **Supplementary Table 2**).

### 2.4 Labelling Surface Antigens

Aliquots of 10^6^ PBMCs were thawed by a 37°C thermostatic bath and washed twice in RPMI 1640 medium (Thermo Fisher Scientific). Samples were processed and stained as described (9) prior to being analyzed by flow cytometry.

### 2.5 *In Vitro* Stimulation and Intracellular Cytokine Staining

Thawed aliquots to analyze intracellular cytokine production were subdivided in three polypropylene tubes. To study cytokine production by monocytes, an aliquot of 3x10^5^ PBMCs was resuspended in 1 mL of RPMI 1640 medium and incubated with 1 mg/mL lipopolysaccharide (from Escherichia coli O111: B4; Merck) in presence of 2 μg/ml Brefeldin A (GolgiPlug, BD Biosciences) and 2.1 μM Monensin (Golgi Stop, BD Biosciences) during 4 hours at 37°C in 5% CO_2_ atmosphere.

To study cytokine production by T and B cells (Except IL-10 producing B cells) an aliquot of 3x10^5^ PBMCs was resuspended and incubated in 1 mL RPMI 1640 medium and stimulated with 50 ng/mL of Phorbol 12‐myristate 13‐acetate (PMA, Merck) and 750 ng/mL Ionomycin (Merck) in presence of 2 μg/ml Brefeldin A and 2.1 μM Monensin during 4 hours at 37°C in 5% CO_2_ atmosphere.

To identify IL-10 producing B cells, an aliquot of 3x10^5^ PBMCs was preincubated in 1 mL RPMI 1640 medium with 3 μg/mL of CpG oligonucleotide (*In vivo*Gen) during 20h at 37°C in 5% CO2 atmosphere. After this, it was stimulated with 50 ng/mL of Phorbol 12‐myristate 13‐acetate (PMA, Merck) and 750 ng/mL Ionomycin (Merck) in presence of 2 μg/ml Brefeldin A and 2.1 μM Monensin during 4 hours at 37°C in 5% CO_2_ atmosphere.

After incubation, the three aliquots were stained with the two-step protocol described previously (9). PBMCs were analyzed in a FACSCanto II flow cytometer (BD Biosciences).

### 2.6 Flow Cytometry

Cells were always analyzed within a maximum period of 1h after staining. Mean autofluorescence values were set using appropriate negative isotype controls. Data analysis was performed using FACSDiva Software V.8.0 (BD Biosciences). A minimum amount of 5x10^4^ events were analyzed. We followed the strategy showed in **Supplementary Figure 1** to identify the different subpopulations. We set a gate including cells with high to intermediate CD45 and low to intermediate side scatter and excluding debris and apoptotic cells. CD4 and CD8 T cells were classified as: naïve (CCR7+ CD45RO−), central memory (CM) (CCR7+ CD45RO+), effector memory (EM) (CCR7− CD45RO+), and terminally differentiated (TD) (CCR7− CD45RO−). Regulatory CD4 T cells (Treg) were defined as CD3+ CD4+ CD25^hi^ CD127^-/low^. CD56 NK cells were classified as: NKT cells (CD3+ CD56^dim^), CD56dim NK cells (CD3- CD56^dim^) and CD56bright NK cells (CD3- CD56^br^). B cells were classified as: naïve (CD19+ CD38^dim^ CD27-), memory (CD19+ CD27^dim^ CD38^dim^), plasmablasts (CD19+ CD27^hi^ CD38^hi^), transitional B cells (CD19+ CD27‐ CD24^hi^ CD38^hi^) cells or regulatory B cells (Breg) (CD19+ IL-10+) cells. PD‐L1 was explored in monocytes by studying its co-expression with CD14 in PBMCs. We also explored intracellular production of IL-1β, IL-6, IL-10, IL-12 and TNFα by monocytes.IL-1β and TNFα represent innate cell activation, IL-12 induces Th1 responses, IL-6 represent innate cell activation and induces Th17 responses and finally, IL-10 is an anti-inflammatory cytokine. We also explored in CD4 and CD8 T cells the production of IFNγ and TNFα, products of Th1 response; IL-17, a product of the Th17 response; GM-CSF, which induces innate cell activation; and IL-10 that has a regulatory function. Finally, we explored B cells producing IL-6, a pro-inflammatory cytokine that induces Th17 cells; TNFα, an inflammatory cytokine; GM-CSF, inducing innate cell activation; and IL-10 a regulatory cytokine. Representative dot plots showing cytokine production by monocytes, B and T cells are shown in **Supplementary Figure 2**.

### 2.7 Flow Cytometry Analyses

We recorded for every leukocyte subset total cell counts per mL of blood, calculated by measuring total lymphocyte and monocyte numbers by a coulter counter, and the percentages of every subset over total mononuclear cells. To avoid bias due to B cell depletion, we also recorded the values of every T, B, NK and monocyte subset relative to total T, B, NK and monocyte cells, respectively.

### 2.8 Immunoglobulin and sNfL Quantification

Serum levels of immunoglobulins (IgG, IgA, and IgM) were measured by nephelometry using a DimensionVista analyzer (Siemens Healthcare Diagnostics) and serum neurofilament light chain (sNfL) levels were measured using the single molecule array (Simoa) NF-light^®^ Assay (Quanterix).

### 2.9 Statistical Analyses

Statistical analyses were performed using GraphPad Prism 8.0 software (GraphPad Prism Inc.). Wilcoxon matched pairs test was used to assess differences between the samples collected at baseline and after 6 months from the same patient. Mann-Whitney-U test was used to compare the subgroups of patients. *P*-values were adjusted using the Bonferroni correction and *p*-values less than 0.05 were considered statistically significant.

The association between NfL and age has been modelled using a Generalized Additive Model for Location, Scale and Shape (GAMLSS) model and age-normalized measures were obtained for each data point. Z score was used as a continuous measure for the number of standard deviations a given datapoint is above/below the mean in samples of healthy controls of the same age.

### 2.10 Ethical Considerations

Written informed consent was obtained from every patient prior to their inclusion in the present study, which was approved by the Ethics Committee of each center participating in this study.

### 2.11 Data Availability Statement

Any anonymized data collected for the purpose of this study will be shared with qualified investigators for 3 years from the initial publication of the study upon reasonable request to the corresponding author.

## 3 Results

Sixty-nine patients with PPMS (53% female) treated with ocrelizumab were included in this study. Age and disease duration [median (range)] were, respectively, 52.0 (33.0-71.0) and 9.2 (1.3-24.1) years and basal EDSS score was 5.5 (1.0-8.0). All patients were followed for 1 year. Fifty-one (73.9%) remained NEDA 1 year after ocrelizumab initiation. Using MRI data collected at baseline, we further classified the patients according to their inflammatory status into Gd+ (at least one Gd-enhancing lesion) and Gd- (no Gd-enhancing lesions) groups. Ten Gd+ (62.5%) and 41 Gd- (77.4%) patients were NEDA at the one-year follow-up. We found no significant differences between the four patient subgroups in terms of baseline clinical characteristics except for the MRI data (**Table 1**).

**Table 1:** Baseline data and patient characteristics.

	All patients (n=69)	Gd+ (n=16)		Gd- (n=53)		p value
		EDA	NEDA	EDA	NEDA	
		(n=6)	(n=10)	(n=12)	(n=41)	
**Age [years] – median (range).**	52.0	51.0	49.0	50.0	54.0	0.204
	(33.0 – 71.0)	(40.0 – 54.0)	(33.0 – 58.0)	(38.0 – 63.0)	(36.0 – 71.0)	
**Sex (F/M).**	37/32	3/3	5/5	6/6	23/18	0.970
**Disease duration [years] – median (range).**	9.2	10.7	7.0	6.6	10.2	0.490
	(1.3 – 24.1)	(2.1 – 15.4)	(1.3 – 13.3)	(1.6 – 19.0)	(1.7 – 24.2)	
**EDSS score – median (range).**	5.5	5.5	6.0	4.0	6.0	0.160
	(1.0 – 8.0)	(3.0 – 8.0)	(3.5 – 6.0)	(1.0 – 6.0)	(2.0 – 7.0)	
**Gd lesions – median (range).**	0.0	1.0	1.0	0.0	0.0	**1.6x10^-14^**
	(0.0 – 4.0)	(1.0 – 3.0)	(1.0 – 4.0)	(0.0 – 0.0)	(0.0 – 0.0)	

F, Female; M, male; EDSS, Expanded Disability Status Scale; n, number of patients; Gd+/-, presence/absence of gadolinium enhancing lesions at baseline; EDA, evidence of disease activity patients at 1 year of follow-up; NEDA, non-evidence of disease activity patients at 1 year of follow-up. Bold values are significant values (p < 0.05).

At baseline, few differences were found between Gd+ and Gd- patients (**Supplementary Figure 3**). sNfL levels were higher in Gd+ group, (p=0.019). Likewise, plasmablast numbers trended to be higher in this group of patients (p=0.029) but significance was lost after Bonferroni correction. By contrast, percentages of B naïve cells with respect to total CD19+ cells were higher (p=0.015) in Gd- patients. No differences were found between those Gd+ and Gd- patients in monocytes, T or NK cell subsets analyzed nor in intracellular cytokine production (data not shown).

We next explored differences in the PBMCs induced by ocrelizumab according to patient group after 6 months of ocrelizumab treatment by addressing its impact on the absolute numbers (**Supplementary Table 2**) and relative percentages (**Supplementary Table 3**) of each cellular subtype.

### 3.1 B Cells

As expected, after 6 months of treatment, ocrelizumab reduced the total numbers of B cells in all groups (**Supplementary Table 2**). After applying the Bonferroni correction, these differences remained statistically significant in the Gd+ NEDA group and the Gd- EDA and NEDA groups (**Figure 1A** and **Supplementary Table 2**). These differences were mainly due to decreased naïve and memory B cell numbers (**Figures 1B, C**, **Supplementary Table 2**). However, there was no statistically significant reduction in any B cell subpopulation in EDA patients in the Gd+ group (**Supplementary Table 2**). This may be partly due to the low number of patients included in this group. However, it should be noted that this patient subgroup had significantly more total (p=0.030) and transitional (p=0.030) B cells than the NEDA patients in the same Gd+ group at the 6-month follow-up (**Figures 1A, D**, respectively; **Supplementary Table 2**). This could also imply a more rapid B cell repopulation in this group. These differences were not observed in the Gd- patients (**Supplementary Table 2**).

**Figure 2:**
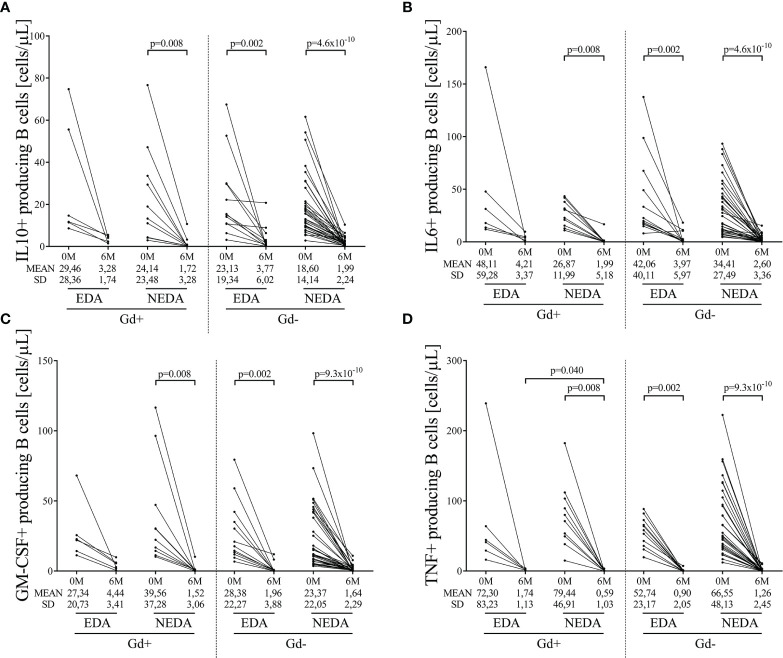
Changes in blood cytokine-producing B cells on ocrelizumab treatment. Cytokine-producing B cells were obtained before (0M) and at 6 months (6M) of ocrelizumab treatment and classified based on their inflammatory status (presence [Gd+] or absence [Gd-] of gadolinium enhancing lesions at baseline) and response (NEDA or EDA) to treatment at one year of follow-up. N was 6, 10, 12 and 41 in Gd+ EDA, Gd+ NEDA, Gd- EDA and Gd- NEDA, respectively. Graphs showing changes in absolute numbers (cells/μL) of IL-10 **(A)**, IL-6 **(B)**, GM-CSF **(C)**, and TNFα **(D)-**producing B cells. IL, interleukin; GM-CSF, granulocyte-macrophage colony-stimulating factor; TNF, tumor necrosis factor alpha; SD, Standard deviation. Bonferroni-corrected p-values are shown.

We next evaluated intracellular cytokine production in B cells. Again, after applying the Bonferroni correction, a drastic reduction in IL-10, IL-6, GM-CSF, and TNFα B cell numbers (**Figures 2A–D**, respectively, **Supplementary Table 2**) was observed in the Gd+ NEDA group and in both Gd- EDA and NEDA patients. However, this was not observed in Gd+ EDA patients, who at 6 months of follow-up showed increased numbers of B cells producing TNFα than NEDA patients of the same group (p=0.04, **Figure 2D** and **Supplementary Table 2**). At 6 months, B cell numbers were very low and thus establishing percentages respective to total B cells with such a small quantity of cells could result highly imprecise. Consequently, we decided not to analyze differences relative to total B cells percentages, contrary to what we did with the rest of the leukocyte populations.

### 3.2 T Cells

#### 3.2.1 Total Cell Counts

We next studied the effect of ocrelizumab in the different T cell subpopulations after 6 months of ocrelizumab treatment. We only found significant differences in the CD20+ T cell subset. It decreased in all groups but after Bonferroni correction differences only remained significant in the Gd+ NEDA group and in both Gd- EDA and NEDA groups (**Figure 1F** and **Supplementary Table 2**). Results were similar when studied separately CD4+ and CD8+ subsets (**Supplementary Figure 4**).

#### 3.2.2 Percentages Relative to CD4+ and CD8+ Subsets

**Figure 3:**
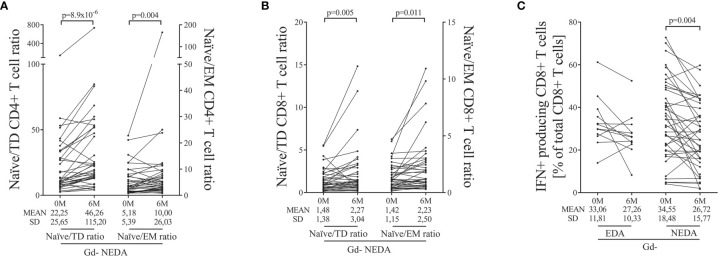
Main changes in blood T cells after ocrelizumab treatment. T cells were obtained before (0M) and at 6 months (6M) of ocrelizumab treatment and classified based on their inflammatory status (presence [Gd+] or absence [Gd-] of gadolinium enhancing lesions at baseline) and response (NEDA or EDA) to treatment at one year of follow-up. N was 6, 10, 12 and 41 in Gd+ EDA, Gd+ NEDA, Gd- EDA and Gd- NEDA, respectively. Graphs showing changes in naïve/effector ratios of CD4 **(A)** and CD8 **(B)** T cells, and changes in percentage of IFNγ-producing CD8 T cells **(C)** relative to total CD8+ T cells. TD, terminally differentiated; EM, effector memory; IFN, gamma-interferon; SD, Standard deviation. Bonferroni-corrected p-values are shown.

No differences were observed in the Gd+ group (**Supplementary Table 3**). However, ocrelizumab treatment modified the T cell compartment in NEDA patients of the Gd- group. We found an increase in the proportion of naïve CD4+ T cells (p=0.004, **Supplementary Table 3**) accompanied by decreases in the percentages of TD (p=0.002, **Supplementary Table 3**) and EM (p=0.041, **Supplementary Table 3**) CD4+ T subsets related to the total CD4+ T cell population. Accordingly, we found increases in the naïve/EM (p=0.004, **Figure 3A**) and naïve/TD (p<0.0001, **Figure 3A**) ratios.

We also found an increase in naïve CD8+ T cell percentages relative to total T CD8+ cells (p=0.031, **Supplementary Table 3**). Although, in this case, it was not associated with significant decreases in effector and TD subpopulations, again, NEDA patients showed an increase in the naïve/EM (p=0.011, **Figure 3B**) and naïve/TD (p=0.005, **Figure 3B**) ratios.

Finally, we studied the relative changes in intracellular cytokine production by CD4+ and CD8+ T cells. No differences were found in cytokine production from CD4+ T cells. However, in the Gd- group, the NEDA patients experienced a decrease in the proportion of CD8+ T cells producing IFNγ (p=0.004, **Figure 3C** and **Supplementary Table 3**) relative to the total CD8+ T cell population.

By contrast, no changes were observed in EDA Gd- group in any T cell subset after six months of ocrelizumab treatment. This suggests that the remodeling of the T cell compartment is important for the response to ocrelizumab in these patients.

### 3.3 Innate Immune Cells

An increase in the total monocyte counts was found in Gd- NEDA patients 6 months after ocrelizumab treatment with the differences being higher in the NEDA group (p=0.034, **Supplementary Table 2**). We next explored if this increase was associated with the total numbers of monocytes producing IL-1, IL-6, IL-10, IL-12, and TNF-alpha or expressing PD-L1. The only significant differences were found in PD-L1+ monocytes. Gd- NEDA patients experienced an increase of the numbers of this subset after ocrelizumab treatment (p=0.013, **Supplementary Table 2**). No significant changes were observed in the proportions of any subset relative to total monocytes. We observed a decrease in the number of CD56bright NK cells (p=0.023, **Supplementary Table 2**) in the Gd- NEDA group after treatment with no variations in the relative proportions of the NK subsets analyzed (**Supplementary Table 3**).

### 3.4 Serum NfL Levels

**Figure 4:**
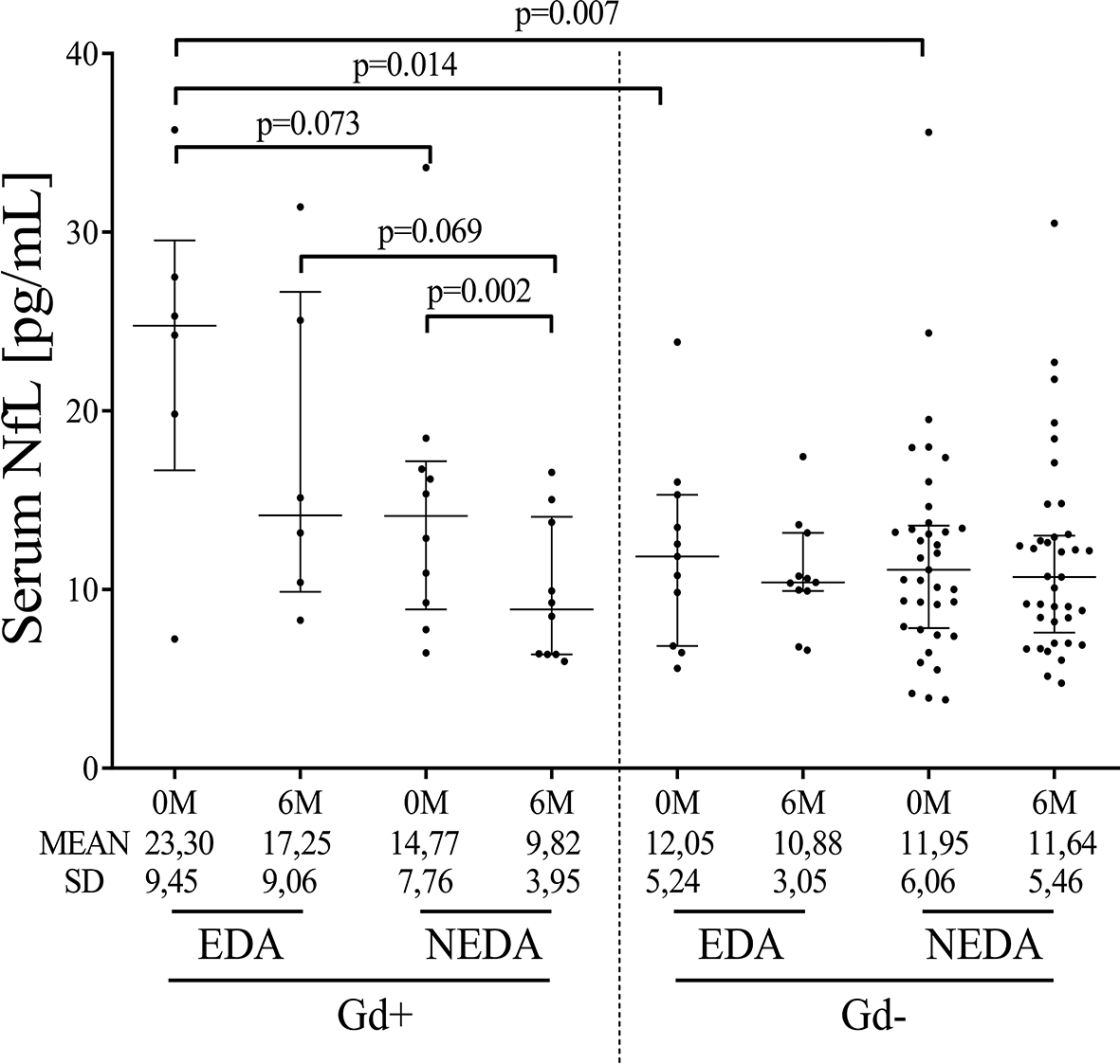
Ocrelizumab treatment induces changes in sNfL levels. sNfL levels (pg/mL) measured before (0M) and at 6 months (6M) of ocrelizumab treatment and classified based on their inflammatory status (presence [Gd+] or absence [Gd-] of gadolinium enhancing lesions at baseline) and response (NEDA or EDA) to treatment at one year of follow-up. N was 6, 10, 12 and 41 in Gd+ EDA, Gd+ NEDA, Gd- EDA and Gd- NEDA, respectively. sNfL, serum neurofilament light-chains; SD, Standard deviation.

Baseline sNfL levels were higher in the Gd+ EDA group compared with the Gd- EDA (p=0.014) and Gd- NEDA (p=0.007) groups (**Figure 4**). They also trended toward higher values than those observed in the Gd+ NEDA patients (p=0.07). Six months after ocrelizumab treatment, sNfL levels were only significantly lower than basal values in the Gd+ NEDA group (**Figure 4**). They did not decrease significantly in the Gd+ EDA patients. The Gd- NEDA and EDA groups, whose baseline sNfL values were not elevated, did not vary significantly from each other upon treatment. When explored the z score normalized by patient age, we observed that in most Gd+ EDA patients, baseline sNfL values were higher than 2 z score levels (median values=2.411) whilst they were mostly between 0 and 2 z score values in most patients from the other three groups (**Table 2**).

**Table 2:** Cohort summary of age-normalized sNfL values.

	0M			6M		
		NfL.zscore			NfL.zscore	
Group	n	Median	IQR	n	Median	IQR
**Gd- NEDA**	41	0.305	[-0.361, 1.495]	41	0.176	[-0.496, 1.175]
**Gd- EDA**	12	0.440	[-0.101, 1.243]	12	0.210	[-0.319, 0.948]
**Gd+ NEDA**	10	1.422	[0.515, 1.642]	10	0.151	[-0.422, 0.812]
**Gd+ EDA**	6	2.411	[2.074, 2.750]	6	1.646	[0.775, 2.395]
**All**	69	0.569	[-0.094, 1.801]	69	0.228	[-0.358, 1.282]

Results of NfL z score are shown as median [25-75% IQR]. Gd+/-, presence/absence of gadolinium enhancing lesions at baseline; EDA, evidence of disease activity patients at 1 year of follow-up; NEDA, non-evidence of disease activity patients at 1 year of follow-up; 0M, 0 Months (pre-ocrelizumab treatment); 6M, 6 Months of ocrelizumab treatment.

### 3.5 Serum Immunoglobulin Values

**Figure 5:**
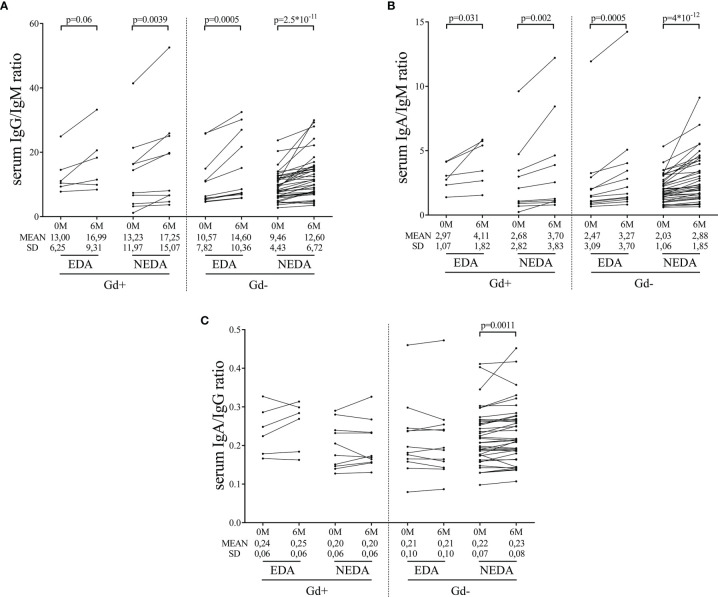
Changes in serum immunoglobulin ratios induced by ocrelizumab treatment. Serum samples were analyzed before (0M) and at 6 months (6M) of ocrelizumab treatment and classified based on their inflammatory status (presence [Gd+] or absence [Gd-] of gadolinium enhancing lesions at baseline) and response (NEDA or EDA) to treatment at one year of follow-up. N was 6, 10, 12 and 41 in Gd+ EDA, Gd+ NEDA, Gd- EDA and Gd- NEDA, respectively. Graphs showing IgG/IgM **(A)**, IgA/IgM **(B)** and IgA/IgG **(C)** ratios. SD, Standard deviation.

We explored changes in serum immunoglobulin concentrations 6 months after the first dose of ocrelizumab. The levels of IgG remained stable, while significant decreases in serum IgM levels were observed in all of them (**Table 3**). Additionally, increased serum IgA levels were observed in the Gd- NEDA group (p=0.006, **Table 3**). However, the most interesting results were observed when the changes in the ratios of the different serum immunoglobulins were explored. The IgA to IgM ratio was augmented in all groups (**Figure 5B**) with these changes highly relevant in Gd- NEDA group (p=4x10^-12^). The IgG to IgM ratio also increased (**Figure 5A**), being again the most prominent changes observed in the Gd- NEDA group (p=2.5x10^-11^). Remarkably, this group of patients experienced an increase in the IgA to IgG ratio (p=0.0011; **Figure 5C**) not observed in any other group.

**Table 3:** Ocrelizumab induced changes in serum immunoglobulin levels.

		EDA (n=18)			NEDA (n=51)		
		0M	6M	*p	0M	6M	*p
**IgG [mg/dL]**	**Gd+**	1113 (795-1573)	1125 (748-1478)	*>0.99*	1095 (744-1120)	995 (737-1133)	*>0.99*
	**Gd-**	880 (750-1023)	927 (791-1010)	*>0.99*	955 (825-1090)	984 (870-1195)	*>0.99*
**IgA [mg/dL]**	**Gd+**	233 (206-324)	237 (210-361)	*>0.99*	179 (138-225)	170 (155-215)	*>0.99*
	**Gd-**	172 (133-196)	177 (144-188)	*>0.99*	199 (139-228)	208 (155-251)	***0.006***
**IgM [mg/dL]**	**Gd+**	72 (63-186)	60 (37-168)	*>0.99*	95 (51-244)	77 (40-157)	***0.018***
	**Gd-**	124 (60-160)	99 (36-134)	***0.009***	102 (80-159)	84 (67-131)	4.8*10^-4^

Results are shown as Median [25-75% IQR]. P values were corrected by using Bonferroni test. Gd+/-, presence/absence of gadolinium enhancing lesions at baseline; EDA, evidence of disease activity patients at 1 year of follow-up; NEDA, non-evidence of disease activity patients at 1 year of follow-up; 0M, 0 Months (pre-ocrelizumab treatment); 6M, 6 Months of ocrelizumab treatment; *p, corrected p value. Bold values are significant values (p < 0.05).

## 4 Discussion

Ocrelizumab selectively depletes CD20+ cells while maintaining B cell reconstitution and pre-existing humoral immunity (11). In patients with PPMS, ocrelizumab not only induces B cell depletion, but reshapes the T cell response toward a low inflammatory profile, resulting in decreased sNfL levels (9).

However, the influence of baseline inflammatory status on these changes has not been fully ascertained. Inflammation is used in the classification of patients with progressive MS. In fact, current guidelines for diagnosing PPMS include two qualifiers: disease activity, defined by MRI or clinical evidence of inflammatory lesions or relapses; and disability progression, defined as a gradually worsening disability independent of relapses (12).

Inflammatory status can also play a role in DMT effectiveness. Overall subgroup analyses evaluating Rituximab in patients with PPMS (OLYMPUS trial) suggested that this drug may affect disease progression in younger patients, particularly those with inflammatory lesions (7). However, results from the ORATORIO trial with ocrelizumab showed that anti-CD20 antibodies are effective in non-inflammatory patients with PPMS (8). This agrees with our results. We explored response to ocrelizumab in a multicenter prospective cohort of 69 patients with PPMS treated with this drug. NEDA patients at 1 year of treatment represented 62% of the inflammatory group (10 out of 16) and 75% of the non-inflammatory one (41 of 53) in our study.

We first explored baseline differences in patients classified according to their inflammatory status (presence [Gd+]/absence [Gd-] of gadolinium-enhancing lesions at baseline). We studied different leukocyte subsets (B cells, T cells, NK cells and monocytes) and sNfL values. As expected, Gd+ patients showed high sNfL values. They also showed a trend to higher plasmablast numbers. The association of plasmablasts with inflammation in MS has been widely documented in the CSF (13). Probably peripheral blood is not the best compartment to study these cells in MS, but our data seem to confirm the role of plasmablasts in inflammation in MS. On the other hand, we found increased values of naïve B cells in Gd- patients. This suggests other function for B cells in non-inflammatory PPMS patients, as antigen presentation. No other differences were found between Gd+ and Gd- patients.

We next studied changes associated with NEDA status in both groups of patients by analyzing T, B, NK, and monocyte cell subsets at baseline and 6 months after receiving the first dose of treatment, before receiving the second one. Patients were classified according to their inflammatory condition (Gd+ or Gd-) and to their response to treatment (NEDA or EDA).

Although B cells clearly diminished in all groups of patients 6 months after the first ocrelizumab dose, in the EDA inflammatory group, total B cell counts were higher at this point. This increase was mainly due to transitional B cells, thus indicating a higher rate of B cell repopulation for these patients, and to TNF-alpha-producing B cells, thus indicating a rapid differentiation into pro-inflammatory B cells. Another characteristic of EDA Gd+ patients was the increased levels of sNfL levels at baseline compared with the non-inflammatory groups, with a trend also observed for higher values than NEDA Gd+ patients. These data show that baseline Gd enhancing lesions and especially high serum neurofilament levels associate in PPMS with a high rate of B cell repopulation and strongly suggest that these patients should benefit from early re-treatment or dose adjustment. By contrast, in NEDA Gd+ patients, the low B cell counts at 6 months were associated with a significant reduction of the sNfL levels 6 months after the first ocrelizumab dose. Finally, in NEDA and EDA Gd- patients, sNfL levels did not change significantly after ocrelizumab treatment, despite the reduction in B cell counts or response to treatment. This is important, since NfL is being proposed as a biomarker for response to different drugs in MS (14) and this could be not the case in patients with low inflammatory activity.

T cell activation and cytokine production are also affected by anti-CD20 DMTs (9, 15). We first studied absolute T cell counts. Only CD20+ T cells decreased in number. This occurred in the four patient groups, with Gd+ EDA being the only one in which the reduction did not reach statistical significance, probably due to the low sample size. The CD20+ T cell subset has been proposed to play an important role in MS pathology because of its highly activated phenotype and proinflammatory and migratory properties (16) and a reduction in these cells, described also for alemtuzumab, fingolimod, and dimethyl fumarate (16), can be beneficial for patients with PPMS treated with ocrelizumab, independent of their baseline inflammatory status.

We next explored the changes in the proportions of the different T cell subsets after 6 months of treatment relative to the total CD4+ and CD8+ T cells. Variations were restricted to the Gd- NEDA group. These patients exhibited a reduction in TD and EM CD4+ T effector cells and increases in naïve CD4+ and CD8+ T cells and in the ratios of naïve/EM and naïve/TD in CD4+ and CD8+ T cells. Likewise, they experienced a decrease in the proportion of CD8+ T cells producing IFNγ. These data show that response to ocrelizumab in Gd- patients is conditioned by reshaping the T cell compartment to a more tolerogenic profile. The activation of the T cell compartment by B cells may play an important role in the pathology of Gd- PPMS. Furthermore, the beneficial effects of different drugs in patients with MS with low inflammatory contribution to their disease may be reflected by changes in other biomarkers aside from sNfL, especially in those with low baseline levels of this protein.

Regarding to Gd- EDA patients, we did not find any clear explanation for the lack of changes in the T cell compartment they showed upon Ocrelizumab treatment. They had no differences on epidemiological, clinical or immune cell subsets at baseline with NEDA group. Exploring antigen presenting B cells and activated dendritic cells in both groups could help to elucidate this conundrum. Future research will demonstrate if antigen presenting B cells could be a biomarker of response to Ocrelizumab in Gd- PPMS patients, as suggested by the remodeling in the T cell compartment showed by NEDA patients.

We also explored changes in innate immune cells at baseline and after 6 months of treatment. Again, we only found significant changes in NEDA Gd- patients. They showed a significant increase in total monocyte counts. This increase was mainly due to PD-L1-expressing monocytes. This molecule is a ligand of the PD-1 receptor, which promotes self-tolerance by suppressing T cell inflammatory activity (17). Its increase has been described in response to other drugs in MS (18). Its up-regulation upon B cell depletion further demonstrates the role of inflammatory B cells in inducing inflammation in cells of the innate immune response and how this can be changed by B cell depletion (19). The up-regulation of PD-L1 expression by monocytes may contribute to the remodeling of the T cell compartment observed in these patients.

Regarding serum immunoglobulins in the four groups of patients, ocrelizumab induced a decrease in serum IgM levels, with no changes in IgG values, as previously reported for patients treated with anti-CD20 antibodies (9, 20). Likewise, all groups of patients showed a decrease in the IgG/IgM and IgA/IgM ratios. Most IgM molecules present in serum are natural antibodies that react against non-protein antigens, anti-lipid specificity being the most frequent (21, 22). Intrathecal synthesis of anti-lipid IgM antibodies associates with an aggressive MS course (22, 23). Thus, the down regulation of the B cells producing these antibodies may have a beneficial effect in MS. Additionally, our data contain interesting results with IgA antibodies. Gd- NEDA patients showed an increase of the levels of this immunoglobulin upon ocrelizumab treatment, and raised values of the ratio IgA/IgG. IgA, produced mostly at mucosal surfaces, functions as a critical mediator of intestinal homeostasis (24) and gut-microbiota reactive IgA plasma cells can migrate to peripheral organs with potential roles in extraintestinal autoimmune diseases (25). In MS, gut microbiota-specific IgA cells are considered a systemic mediator of the disease behaving as an informative biomarker during active neuroinflammation (26). In the experimental model of the disease, migration of IgA-producing plasma cells from the intestinal mucosa to the CNS has proven to down-modulate disease activity. This was attributed to IL-10 production in these cells (27), but often, natural IgA antibodies produced by plasma cells of the gut mucosa recognize similar antigens that natural IgM antibodies present in serum (28, 29). These IgA antibodies could block antigens recognized by IgM, thus avoiding complement fixation and diminishing axonal damage.

In summary this study shows that baseline inflammation could determine the immunological pathways that drive the response to Ocrelizumab in PPMS and that, regardless of baseline MRI activity, B cell depletion with ocrelizumab can modify both underlying mechanisms, and be effective in more than 60% of patients.

## Data Availability Statement

The original contributions presented in the study are included in the article/**Supplementary Material**. Further inquiries can be directed to the corresponding author.

## Ethics Statement

The studies involving human participants were reviewed and approved by Ethics Committee of each participating hospital. The patients/participants provided their written informed consent to participate in this study.

## Author Contributions

JF-V drafted the manuscript, major role in performing experiments, acquisition and analysis of data. PW-D, PL-S, and NV collected samples and performed flow cytometry experiments. JK and AM contributed to sNfL measurement. ER-M and ER supervised flow cytometry studies. EM, VM-L, JM-L, GI, CO-G, FG-G, SS, AS, YB, CD-P, GV-L, JD-D, YA, LB, CÍ, IG-S, LR, JG-D, JS, SL, JM, and LC-F visited MS patients, contributed by sending samples or collected clinical data. LV designed and supervised the study. All authors revised the manuscript and approved the final version.

## Funding

This work was supported by Red Española de Esclerosis Múltiple (REEM) (RD16/0015/0001; RD16/0015/0002; RD16/0015/0003; RD16/0015/0008; RD16/0015/0013) and PI18/00572 integrated in the Plan Estatal I+D+I and co-funded by ISCIII-Subdirección General de Evaluación and Fondo Europeo de Desarrollo Regional (FEDER, “Una manera de hacer Europa”).

## Conflict of Interest

EM received research grants, travel support or honoraria for speaking engagements from Biogen, Merck, Novartis, Roche, and Sanofi-Genzyme; JK received speaker fees, research support, travel support, and/or served on advisory boards by ECTRIMS, Swiss MS Society, Swiss National Research Foundation (320030_189140/1), University of Basel, Bayer, Biogen, Celgene, Merck, Novartis, Roche, Sanofi; VM-L received grants and consulting or speaking fees from Almirall, Biogen, Celgene, Genzyme, Merck, Novartis, Roche and Teva; JM-L has received grants and consulting or speaking fees from Almirall, Biogen, Bristol-Myers-Squibb, Genzyme, Merck, Novartis, Roche and Teva; GI has received consultancy/advice and Conference- travel support from Bayer, Novartis, Sanofi, Merck Serono, Roche, Actelion Celgene and Teva; CO-G has received speaker and consulting fees from Biogen, Celgene, Merck KGaA (Darmstadt, Germany), Novartis, Roche, Sanofi Genzyme and Teva; FG-G has received funding for research grants, travel support and honoraria for speaking engagements from: Bayer, Biogen, Roche, Merck, Novartis, Almirall, Teva and Genzyme-Sanofi; SS received research grants, travel support, or honoraria for speaking engagements from Almirall, Bayer, Biogen, Merck, Mylan, Novartis, Roche, Sanofi-Genzyme and Teva; AS reports compensation for consulting services and speaker honoraria from Merck-Serono, Biogen-Idec, Sanofi-Aventis, Teva Pharmaceutical Industries Ltd, Novartis, Roche, and Alexion; YB received speaking honoraria from Biogen, Novartis and Genzyme; LB received funding for research projects or in the form of conference fees, mentoring, and assistance for conference attendance from: Bayer, Biogen, Roche, Merk, Novartis, Almirall, Celgen and Sanofi; IG-S received research grants, travel support and honoraria for speaking engagements from Biogen, Merck, Novartis, Roche, Sanofi-Genzyme, TEVA and Alexion; LR received travel support, and honoraria for speaking engagements from Biogen, Merck, Roche and Sanofi-Genzyme; JS received funding for research projects and conference fees, mentoring, and assistance for conference attendance from: Teva, Merck, Biogen, Roche, Novartis, and Sanofi; SL received compensation for consulting services and speaking honoraria from Merck-Serono, Biogen-Idec, Sanofi-Aventis, Teva Pharmaceutical Industries Ltd, Novartis and Roche; LC-F received speaker fees, travel support, and/or served on advisory boards by Biogen, Sanofi, Merck, Bayer, Novartis, Roche, Teva, Celgene, Ipsen, Biopas, Almirall; LV received research grants, travel support or honoraria for speaking engagements from Biogen, Merck, Novartis, Roche, Sanofi-Genzyme and Bristol-Myers.

The remaining authors declare that the research was conducted in the absence of any commercial or financial relationships that could be construed as a potential conflict of interest.

## Publisher’s Note

All claims expressed in this article are solely those of the authors and do not necessarily represent those of their affiliated organizations, or those of the publisher, the editors and the reviewers. Any product that may be evaluated in this article, or claim that may be made by its manufacturer, is not guaranteed or endorsed by the publisher.
