# Supplementary material for: Baseline Inflammatory Status Reveals Dichotomic Immune Mechanisms Involved In Primary-Progressive Multiple Sclerosis Pathology
Source: Front Immunol. 2022 Mar 21;13:842354. doi: 10.3389/fimmu.2022.842354 (PMC8977599; doi:10.3389/fimmu.2022.842354)

**Supplementary Figure 1.** **Gating strategy with representative images for flow cytometry analysis.**

Footnote to Supplementary Figure 1: Total events were first gated to exclude debris and apoptotic cells **(A, gate P1)** and then gated for doublet discrimination **(B, gate P2)**. Cells were further analyzed to identify leukocytes **(C, gate P3)** for their CD45 staining, including monocytes **(C, gate Mon)** and lymphocytes **(C, gate Lymph)**. Expression of PD-L1 and CD14 was used to gate PD-L1-expressing monocytes **(D, gate PD-L1+ Mon)**.We gated B cells for their CD19 expression **(E, gate CD19)**. CD27 and CD38 expression identified memory B cells **(J, gate memory B)**, plasmablasts **(J, gate plasmabl)**, naïve B cells **(J, gate naïve B)** and CD27- CD38bright cells **(J, gate CD27- CD38++)**. Transitional B cells **(K, gate transitional B)** were identified for the expression of CD24 in the CD27- CD38bright subset. Expression of CD3 and CD20 identified CD20+ T cells **(F, gate CD20+ T)**. Expression of CD3 and CD56 identified NKT **(G, gate NKT)**, CD56dim NK **(G, gate 56dim)** and CD56bright NK **(G, gate 56br)** cells. Expression of CD3 and CD4 identified total CD4+ **(H, gate CD4+ T)** T cells and CD3 and CD8 ones identified total CD8+ **(I, gate CD8+ T)** T cells. According to their CCR7 and CD45RO expression we identified naïve, central memory (CM), effector memory (EM) and terminally differentiated (TD) subsets of CD4+ and CD8+ T cells **(M and N,** respectively). Additionally, we identified CD4+ regulatory T cells **(L, gate CD4+ Treg)** using their CD25 and CD127 expression.


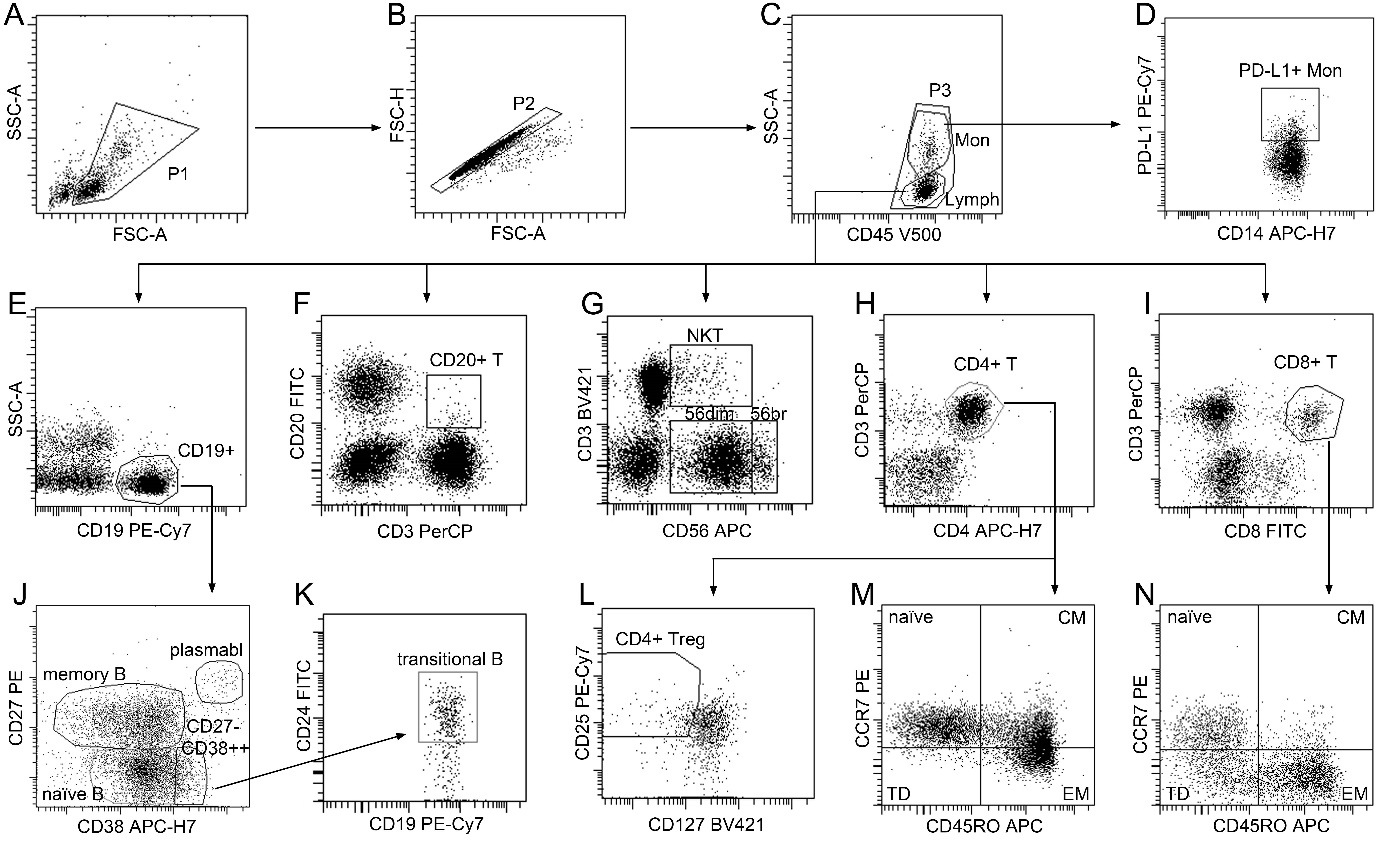

Supplement: Supplementary file 1 [file DataSheet_1.docx]
