# Supplementary material for: Baseline Inflammatory Status Reveals Dichotomic Immune Mechanisms Involved In Primary-Progressive Multiple Sclerosis Pathology
Source: Front Immunol. 2022 Mar 21;13:842354. doi: 10.3389/fimmu.2022.842354 (PMC8977599; doi:10.3389/fimmu.2022.842354)

**Supplementary Figure 2.** **Representative flow cytometry dot plots of non-stimulated and stimulated PBMCs.**

Footnote to Supplementary Figure 2: **(A)** Representative images of non-stimulation (non-stimulated) controls and, for comparison, of LPS stimulated (stimulated) monocytes producing tumor necrosis factor alpha (TNFa), interleukin (IL)-1beta (IL1B), IL10, IL6 and IL12. Percentages are referred to total CD14+ monocytes. **(B)** Representative images of non-stimulation (non-stimulated) controls and, for comparison, of phorbol-myristate-acetate (PMA) and ionomycin stimulated (stimulated) CD4+ and CD8+ T lymphocytes producing TNFa, Interferon gamma (IFNg), IL10, granulocyte macrophage-colony stimulating factor (GM-CSF) and IL17. Percentages are referred to total CD4+ or CD8+ T cells, respectively. **(C)** Representative images of non-stimulation (non-stimulated) controls and, for comparison, of CpG-ODNs overnight cultured and PMA and Ionomycin stimulated (stimulated) B lymphocytes producing TNFa, IL6, IL10 and GM-CSF. Percentages are referred to total CD19+ B cells.


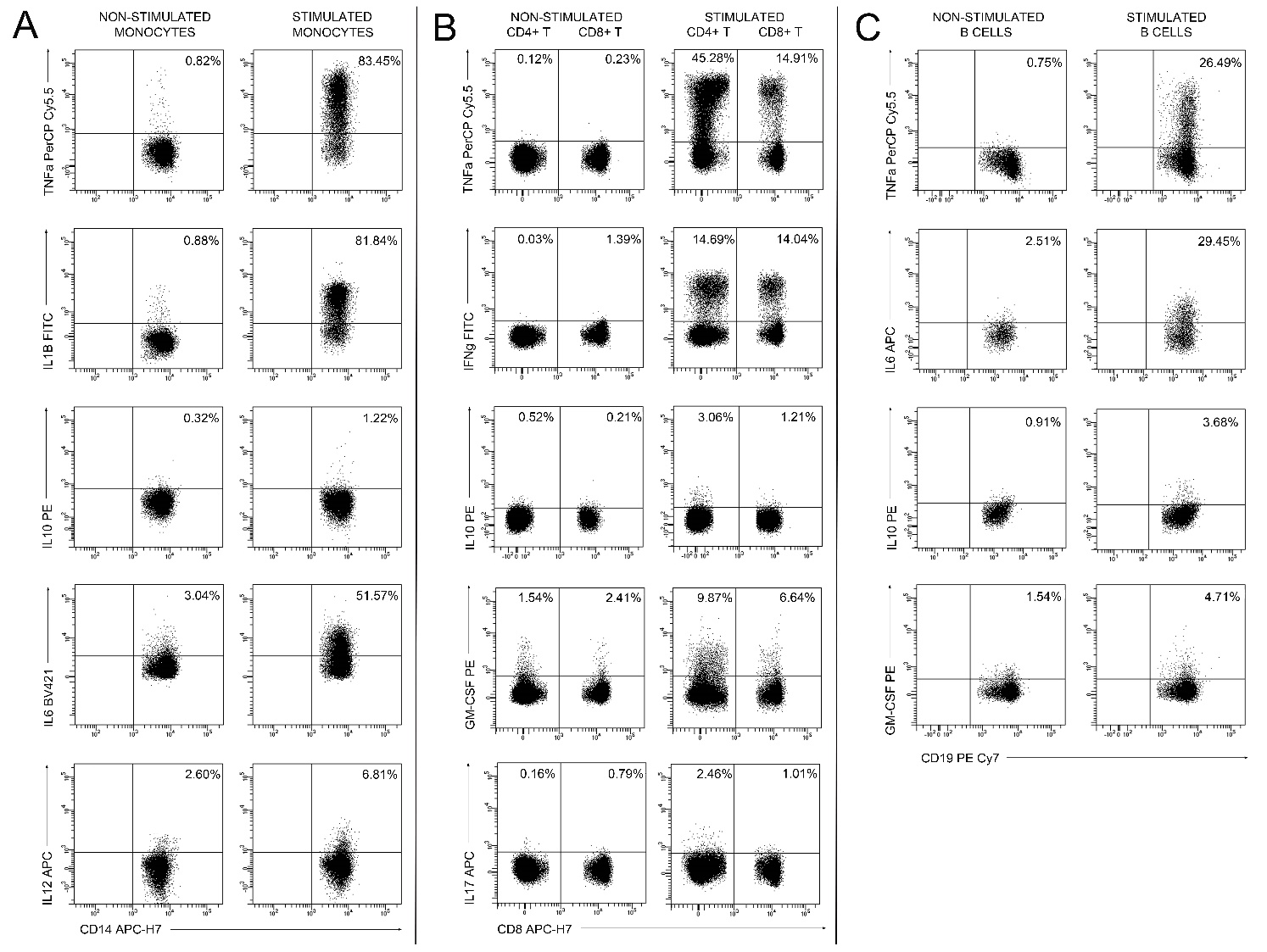

Supplement: Supplementary file 2 [file DataSheet_2.docx]
