# Supplementary material for: Baseline Inflammatory Status Reveals Dichotomic Immune Mechanisms Involved In Primary-Progressive Multiple Sclerosis Pathology
Source: Front Immunol. 2022 Mar 21;13:842354. doi: 10.3389/fimmu.2022.842354 (PMC8977599; doi:10.3389/fimmu.2022.842354)

**Supplementary Figure 3.** Main differences based on baseline inflammatory patient status.

Footnote to Supplementary Figure 3: Baseline differences between inflammatory (presence [Gd+, n=16] of gadolinium enhancing lesions) and non-inflammatory PPMS patients (abscense [Gd-, n=53] of gadolinium enhancing lesions). Graphs showing changes in **(A)** serum neurofilament light chains (NfL) levels and **(B)** percentages of transitional (Tran B), naïve, memory (Mem B) B cells, and plasmablasts (Pb), referred to total CD19+ B cells**.** Median and 25%–75% interquartile range values are shown. SD, Standard deviation. Bonferroni-corrected p-values are shown.


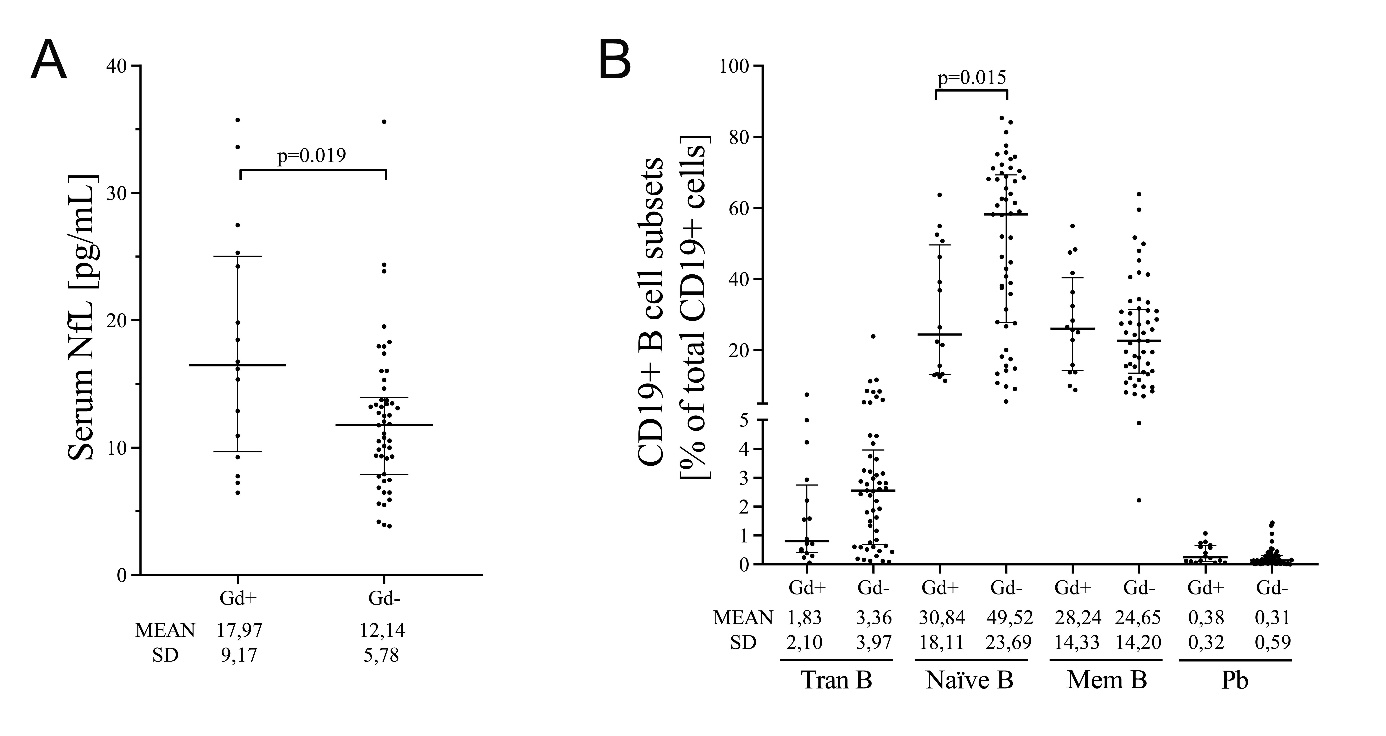

Supplement: Supplementary file 3 [file DataSheet_3.docx]
