# Supplementary material for: Baseline Inflammatory Status Reveals Dichotomic Immune Mechanisms Involved In Primary-Progressive Multiple Sclerosis Pathology
Source: Front Immunol. 2022 Mar 21;13:842354. doi: 10.3389/fimmu.2022.842354 (PMC8977599; doi:10.3389/fimmu.2022.842354)

**Supplementary Figure 4.** **Changes in blood CD20 T cell subsets induced by ocrelizumab.**

Footnote to Supplementary Figure 4: CD20 T cell subsets were obtained before (0M) and at 6 months (6M) of ocrelizumab treatment and classified based on their inflammatory status (presence [Gd+] or absence [Gd-] of gadolinium enhancing lesions at baseline) and response (NEDA or EDA) to treatment at one year of follow-up. N was 6, 10, 12 and 41 in Gd+ EDA, Gd+ NEDA, Gd- EDA and Gd- NEDA, respectively. Graphs showing changes in absolute numbers (cells/μL) of CD4+ CD20+ T cells **(A)** and CD8+ CD20+ T cells **(B).** Median and 25%–75% interquartile range values are shown. SD, Standard deviation. Bonferroni-corrected p-values are shown.


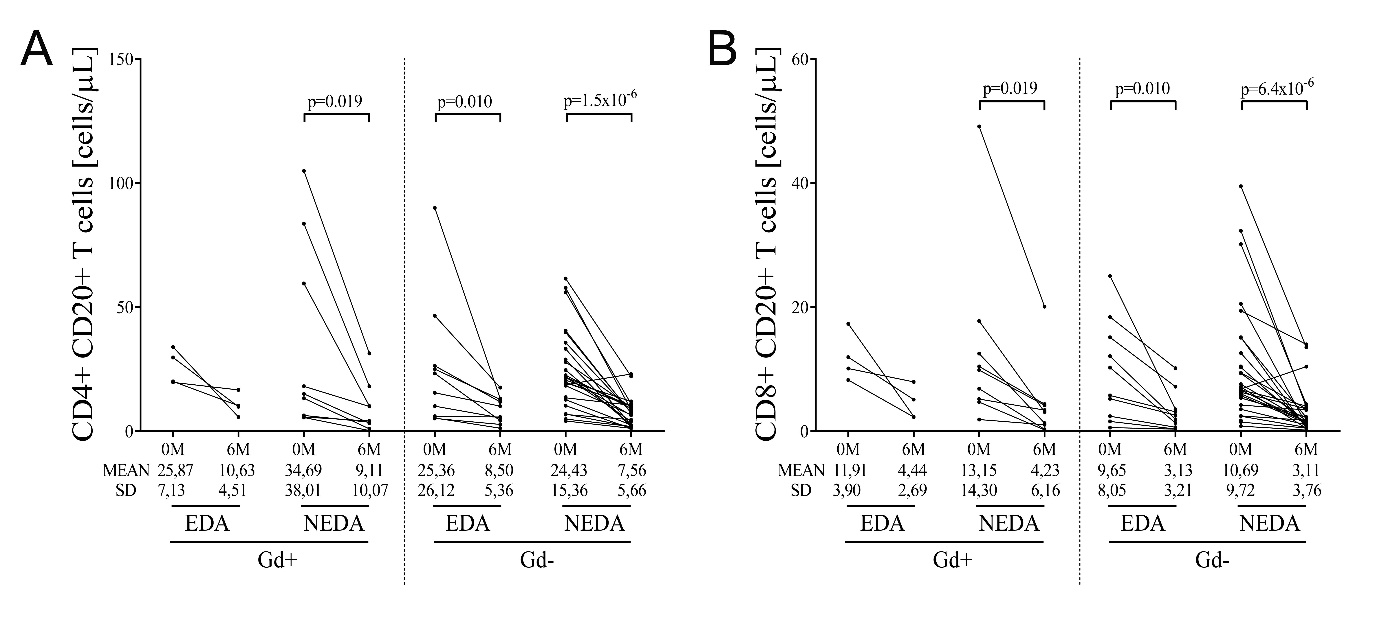

Supplement: Supplementary file 4 [file DataSheet_4.docx]
