# Supplementary material for: Baseline Inflammatory Status Reveals Dichotomic Immune Mechanisms Involved In Primary-Progressive Multiple Sclerosis Pathology
Source: Front Immunol. 2022 Mar 21;13:842354. doi: 10.3389/fimmu.2022.842354 (PMC8977599; doi:10.3389/fimmu.2022.842354)
Supplement: Supplementary file 5 [file Table_1.docx]

**Supplementary Table 1.** Monoclonal antibodies used in this study.

| ***Monoclonal antibody*** | ***Clone*** | ***Conjugated Fluorochrome*** | ***Manufacturer*** |
| --- | --- | --- | --- |
| **CD8** | *SK1* | *FITC* | BD Biosciences |
| **CD20** | *L27* |  |  |
| **CD24** | *ML5* |  |  |
| **Interferon-gamma (IFNγ)** | *4S.B3* |  |  |
| **Interleukin (IL)-1β** | *AS10* |  |  |
| **CD27** | *M-T271* | *PE* | BD Biosciences |
| **CD197 (CCR7)** | 150503 |  |  |
| **IL-10** | JES3-19F1 |  |  |
| **Granulocyte-macrophage colony stimulation factor (GM-CSF)** | BVD2-21C11 |  |  |
| **CD3** | *SK7* | *PerCP Cy5.5* | BD Biosciences |
| **Tumor necrosis factor-alpha (TNFα)** | MAb11 |  |  |
| **CD19** | *SJ25C1* | *PE Cy7* | BD Biosciences |
| **CD25** | M-A251 |  |  |
| **Programmed death‐ligand (PD‐L1)** | MIH1 |  |  |
| **CD45RO** | *UCHL1* | *APC* | BD Biosciences |
| **CD56** | *NCAM16.2* |  |  |
| **IL-6** | *MQ2-13A5* |  |  |
| **IL-12** | *C11.5* |  |  |
| **IL-17** | 41809 | *APC* | R&D Systems |
| **CD4** | *RPA-T4* | *APC-H7* | BD Biosciences |
| **CD8** | *SK1* |  |  |
| **CD14** | *MφP9* |  |  |
| **CD38** | *HB7* |  |  |
| **CD3** | *UCHT1* | *BV421* | BD Biosciences |
| **CD127** | HIL-7R-M21 |  |  |
| **IL-6** | MQ2-13A5 |  |  |
| **CD45** | *HI30* | *V500* | BD Biosciences |
