# Supplementary material for: Baseline Inflammatory Status Reveals Dichotomic Immune Mechanisms Involved In Primary-Progressive Multiple Sclerosis Pathology
Source: Front Immunol. 2022 Mar 21;13:842354. doi: 10.3389/fimmu.2022.842354 (PMC8977599; doi:10.3389/fimmu.2022.842354)
Supplement: Supplementary file 6 [file Table_2.docx]

**Supplementary Table 2.** Ocrelizumab induced changes in leukocyte blood subset absolute numbers.

Footnote to Supplementary Table 2: Results are shown as Median [25-75% IQR]. Absolute numbers are displayed as cells/µL. P values were corrected by using Bonferroni test. Gd+/-, presence/absence of gadolinium enhancing lesions at baseline; EDA, evidence of disease activity patients at 1 year of follow-up; NEDA, non-evidence of disease activity patients at 1 year of follow-up; 0M, 0 Months (pre-ocrelizumab treatment); 6M, 6 Months of ocrelizumab treatment; *p, corrected p value; IL, interleukin; GM-CSF, Granulocyte-Macrophage Colony-Stimulating Factor; TNFα, Tumor Necrosis Factor-alpha; Treg, regulatory T cells; CM, Central Memory; EM, Effector Memory; TD, Terminally differentiated; IFNγ, Interferon gamma; PD-L1, Programmed Death-ligand 1; NKT, Natural Killer T cells.

|  | **Gd+(n=16)** | | | | | | **Gd- (n=53)** | | | | | |
| --- | --- | --- | --- | --- | --- | --- | --- | --- | --- | --- | --- | --- |
| **ABSOLUTE NUMBERS**  **(n=69)** | **EDA (n=6)** | | | **NEDA (n=10)** | | | **EDA (n=12)** | | | **NEDA (n=41)** | | |
|  | **0M** | **6M** | ***p** | **0M** | **6M** | ***p** | **0M** | **6M** | ***p** | **0M** | **6M** | ***p** |
| **B cells** | 149.2  [106.8-226.1] | 28.3  [20.0-78.1] | 0.188 | 297.3  [193.2-382.6] | 2.31  [1.5-7.5] | **0.012** | 231.1  [129.9-318.3] | 5.1  [1.2-56.9] | **0.003** | 226.9  [153.9-298.9] | 7.6  [2.5-34.1] | **6.9*10^-10^** |
| **Traitional B** | 4.0  [3.3-16.7] | 12.9  [7.4-49.0] | >0.99 | 5.0  [1.7-6.8] | 0.1  [0.1-1.7] | 0.504 | 6.9  [2.9-21.4] | 0.5  [0.2-18.9] | >0.99 | 4.4  [2.5-10.7 ] | 1.6  [0.1-14.0] | >0.99 |
| **Naïve B** | 90.8  [65.3-131.8] | 5.9  [1.7-15.8] | 0.188 | 158.1  [116.0-204.9] | 0.2  [0.1-0.5] | **0.012** | 128.3  [88.6-166.6] | 0.7  [0.1-5.2] | **0.003** | 159.7  [90.9-199.3] | 0.5  [0.2-3.9] | **6.9*10^-10^** |
| **Memory B** | 36.2  [20.7-84.6] | 4.1  [1.9-9.0] | 0.188 | 87.6  [42.1-144.4] | 1.2  [0.7-2.5] | **0.012** | 45.0  [23.5-119.0] | 1.7  [0.5-4.6] | **0.003** | 37.9  [27.6-84.4] | 2.4  [1.1-5.0] | **6.9*10^-10^** |
| **Plasmablasts** | 0.8  [0.5-1.2] | 0.7  [0.2-1.4] | >0.99 | 0.6  [0.2-1.2] | 0.2  [0.2-0.3] | 0.633 | 0.7  [0.2-1.1] | 0.1  [0.0-0.5] | 0.097 | 0.2  [0.1-0.5] | 0.2  [0.0-0.6] | >0.99 |
| **IL10+ B** | 13.2  [10.8-60.4] | 3.2  [1.5-4.9] | 0.157 | 16.1  [4.3-37.0] | 0.4  [0.2-1.5] | **0.008** | 15.2  [10.8-29.9] | 1.3  [0.3-5.5] | **0.002** | 15.6  [9.6-28.6] | 1.2  [0.4-3.2] | **4.6*10^-10^** |
| **IL6+ B** | 24.7  [13.3-77.3] | 4.4  [1.2-6.3] | 0.157 | 26.7  [14.8-39.0] | 0.2  [0.1-0.9] | **0.008** | 21.4  [16.8-63.0] | 1.0  [0.2-8.6] | **0.004** | 29.1  [11.1-47.2] | 1.2  [0.2-3.9] | **4.6*10^-10^** |
| **GMCSF+ B** | 22.4  [13.5-36.3] | 4.2  [1.5-7.0] | 0.157 | 26.2  [13.6-59.5] | 0.6  [0.2-1.0] | **0.008** | 19.4  [12.2-40.2] | 0.4  [0.1-0.8] | **0.002** | 15.0  [8.2-42.4] | 0.8  [0.3-2.4] | **9.3*10^-10^** |
| **TNFα+ B** | 42.7  [25.9-107.7] | 1.5  [0.9-2.7] | 0.157 | 75.6  [46.6-105.6] | 0.2  [0.1-0.6] | **0.008** | 55.7  [32.0-70.9] | 0.2  [0.1-0.9] | **0.002** | 49.7  [33.1-99.6] | 0.3  [0.1-1.6] | **9.3*10^-10^** |
| **CD20 T cells** | 35.0  [30.4-48.0] | 13.8  [9.1-22.3] | 0.625 | 28.0  [11.7-83.1] | 7.6  [3.0-18.3] | **0.023** | 25.8  [10.2-48.0] | 9.7  [5.0-18.3] | **0.012** | 30.0  [17.7-44.0] | 8.9  [3.2-13.0] | **8.9*10^-7^** |
| **CD4 T cells** | 605.0  [456.0-805.3] | 595.7  [547.9-958.8] | >0.99 | 837.3  [672.4-1259.0] | 791.8  [719.2-1292.0] | >0.99 | 821.2  [447.1-1026.0] | 945.1  [777.9-1400.0] | 0,210 | 792.2  [643.3-1204.0] | 986.4  [677.4-1359.0] | >0.99 |
| **Treg CD4** | 76.1  [53.6-104.6] | 92.7  [75.9-105.0] | >0.99 | 107.5  [74.2-139.6] | 105.2  [97.5-128.1] | >0.99 | 58.9  [45.9-122.9] | 103.0  [70.1-141.3] | 0.923 | 92.2  [64.7-116.3] | 98.7  [69.7-131.2] | >0.99 |
| **Naïve CD4** | 235.0  [108.3-406.2] | 253.0  [193.2-396.0] | >0.99 | 397.1  [230.2-785.4] | 347.4  [275.7-769.4] | >0.99 | 425.0  [191.0-624.7] | 518.0  [353.9-992.0] | 0.122 | 437.0  [248.7-686.4] | 570.1  [313.3-823.8] | 0,280 |
| **CM CD4** | 214.2  [164.0-278.0] | 217.2  [198.2-345.2] | >0.99 | 239.7  [154.5-291.9] | 246.2  [173.9-322.4] | >0.99 | 170.7  [104.5-333.6] | 246.7  [176.7-308.5] | >0.99 | 260.7  [149.3-312.4] | 245.7  [176.1-353.2] | >0.99 |
| **EM CD4** | 127.3  [58.2-240.7] | 132.5  [101.5-219.1] | >0.99 | 161.3  [51.7-213.8] | 124.1  [79.0-196.7] | >0.99 | 101.6  [62.3-210.0] | 131.7  [88.4-204.6] | >0.99 | 117.3  [76.4-149.2] | 119.7  [65.9-179.8] | >0.99 |
| **TD CD4** | 14.8  [6.6-31.8] | 17.8  [11.3-30.0] | >0.99 | 33.1  [17.0-78.6] | 24.7  [13.4-53.8] | >0.99 | 23.1  [10.6-74.2] | 39.5  [12.5-80.2] | >0.99 | 27.4  [17.5-34.0] | 24.9  [14.2-37.6] | >0.99 |
| **IL10+ CD4** | 10.3  [6.4-13.3] | 11.2  [7.4-18.3] | >0.99 | 14.6  [8.1-21.3] | 11.8  [10.0-14.4] | >0.99 | 11.3  [5.2-19.0] | 17.2  [7.1-24.0] | >0.99 | 10.6  [4.1-20.5] | 12.4  [7.1-26.4] | >0.99 |
| **GMCSF+ CD4** | 57.1  [42.5-66.1] | 68.2  [54.9-92.7] | >0.99 | 110.5  [51.9-204.4] | 106.4  [62.3-149.8] | >0.99 | 61.4  [44.7-111.1] | 96.3  [69.6-133.4] | >0.99 | 76.8  [47.1-144.3] | 80.5  [38.8-145.6] | >0.99 |
| **TNFα+ CD4** | 415.7  [303.9-594.2] | 516.1  [438.0-709.1] | 0,938 | 494.5  [281.4-723.2] | 439.1  [295.5-648.0] | >0.99 | 553.8  [349.6-661.0] | 619.0  [440.2-770.8] | >0.99 | 480.4  [363.7-728.3] | 569.0  [348.4-865.3] | 0,864 |
| **IL17+ CD4** | 7.2  [5.8-15.6] | 9.5  [7.8-11.5] | >0.99 | 9.5  [8.2-17.4] | 14.8  [9.4-20.1] | 0,840 | 10.1  [3.9-13.0] | 16.1  [8.2-40.5] | >0.99 | 12.4  [8.1-17.8] | 13.7  [7.5-25.2] | >0.99 |
| **IFNγ+ CD4** | 84.8  [54.3-179.0] | 97.4  [55.1-180.5] | >0.99 | 108.1  [84.3-132.7] | 97.7  [88.4-149.7] | >0.99 | 90.7  [31.2-167.9] | 82.4  [50.5-112.1] | >0.99 | 82.4  [48.5-134.1] | 72.2  [38.5-131.1] | >0.99 |
| **CD8 T cells** | 280.7  [203.5-325.9] | 246.7  [188.4-328.5] | >0.99 | 306.1  [202.4-620.3] | 302.8  [220.5-674.0] | >0.99 | 242.9  [134.6-351.2] | 241.0  [125.8-312.7] | >0.99 | 242.5  [163.0-355.2] | 231.9  [138.2-333.9] | >0.99 |
| **Naïve CD8** | 68.9  [30.9-126.8] | 69.7  [39.3-116.0] | >0.99 | 76.7  [44.1-158.4] | 70.9  [60.2-178.7] | >0.99 | 86.3  [35.8-120.2] | 91.6  [45.7-120.1] | >0.99 | 57.5  [43.9-109.6] | 68.9  [42.0-122.3] | >0.99 |
| **CM CD8** | 18.9  [11.3-28.0] | 19.6  [7.9-32.7] | >0.99 | 14.7  [8.6-26.2] | 24.2  [7.4-37.2] | 0,645 | 8.7  [5.2-30.7] | 12.1  [4.0-22.4] | >0.99 | 16.8  [12.0-44.4] | 16.5  [8.9-29.3] | >0.99 |
| **EM CD8** | 85.6  [38.2-114.7] | 68.2  [50.8-97.9] | >0.99 | 91.3  [47.5-151.3] | 75.8  [48.4-120.6] | >0.99 | 42.8  [24.3-110.0] | 47.3  [11.9-80.3] | >0.99 | 67.5  [35.5-107.3] | 50.8  [28.0-102.1] | 0,358 |
| **TD CD8** | 80.6  [43.7-126.3] | 67.4  [47.5-133.5] | >0.99 | 134.3  [26.5-285.4] | 126.4  [28.0-286.8] | >0.99 | 82.9  [50.3-111.3] | 65.9  [33.9-146.3] | >0.99 | 65.7  [27.3-143.8] | 50.7  [28.6-109.2] | 0.739 |
| **IL10+ CD8** | 8.1  [4.5-15.1] | 11.6  [8.3-15.9] | >0.99 | 16.7  [5.8-24.5] | 9.2  [7.7-11.2] | >0.99 | 5.0  [1.1-10.3] | 11.3  [2.8-15.5] | 0,161 | 4.0  [2.0-11.0] | 5.1  [2.5-12.0] | >0.99 |
| **GM-CSF+ CD8** | 29.0  [14.1-47.5] | 29.7  [17.9-50.9] | >0.99 | 57.6  [13.1-81.4] | 45.1  [17.2-110.6] | >0.99 | 18.5  [12.1-33.0] | 28.8  [12.7-53.2] | >0.99 | 27.7  [10.0-41.3] | 19.8  [11.1-40.5] | >0.99 |
| **TNFα+ CD8** | 119.0  [77.9-167.7] | 147.6  [101.3-190.0] | >0.99 | 170.3  [102.0-239.9] | 195.1  [121.8-293.4] | >0.99 | 138.2  [51.9-201.6] | 101.2  [72.6-166.9] | >0.99 | 107.0  [65.7-183.8] | 88.2  [59.6-175.7] | >0.99 |
| **IL17+ CD8** | 7.6  [6.1-12.3] | 7.8  [5.5-11.2] | >0.99 | 5.2  [3.4-24.9] | 8.4  [4.8-18.1] | >0.99 | 4.3  [2.7-10.5] | 8.2  [3.8-16.2] | >0.99 | 6.1  [3.0-10.8] | 8.1  [2.8-15.9] | >0.99 |
| **IFNγ+ CD8** | 79.2  [47.4-138.3] | 84.9  [44.8-153.5] | >0.99 | 120.7  [57.2-223.2] | 129.8  [83.3-183.0] | >0.99 | 97.2  [37.0-133.9] | 65.6  [50.7-122.4] | >0.99 | 81.3  [30.3-135.1] | 54.0  [25.4-88.5] | 0,241 |
| **Monocytes** | 308.7  [254.5-539.5] | 403.9  [314.1-538.9] | >0.99 | 381.3  [242.9-507.6] | 544.3  [309.4-645.2] | >0.99 | 383.5  [245.9-497.5] | 413.3  [256.9-550.3] | >0.99 | 360.0  [303.7-477.9] | 493.7  [407.9-771.8] | **0.034** |
| **PD-L1 Mon** | 26.4  [15.5-132.2] | 70.4  [17.3-166.6] | 0,500 | 34.4  [15.9-56.8] | 42.7  [22.8-78.6] | 0,336 | 19.6  [8.4-40.3] | 15.5  [10.5-40.2] | >0.99 | 20.2  [6.8-57.6] | 39.6  [8.2-73.9] | **0.013** |
| **IL1B+ Mon** | 5.2  [2.0-51.2] | 23.2  [2.4-31.8] | >0.99 | 6.6  [2.6-15.9] | 6.7  [4.3-10.8] | >0.99 | 2.5  [1.5-15.1] | 7.7  [4.5-16.2] | >0.99 | 9.3  [3.5-24.1] | 12.5  [3.4-31.4] | 0,370 |
| **IL10+ Mon** | 12.0  [4.2-31.5] | 12.7  [5.0-36.8] | >0.99 | 6.8  [3.6-18.0] | 10.0  [7.4-15.8] | 0.137 | 6.9  [3.5-11.6] | 7.5  [3.2-15.6] | >0.99 | 9.5  [3.8-19.5] | 8.3  [4.3-27.4] | >0.99 |
| **TNF+ Mon** | 25.2  [5.3-30.8] | 13.0  [8.0-37.2] | >0.99 | 14.9  [10.3-21.3] | 11.8  [8.6-22.2] | >0.99 | 10.8  [4.0-18.5] | 13.1  [8.7-22.5] | >0.99 | 11.9  [6.0-29.3] | 21.6  [6.1-48.4] | 0,271 |
| **IL12+ Mon** | 12.3  [7.2-96.5] | 50.2  [7.7-94.2] | >0.99 | 17.9  [9.5-38.1] | 39.2  [16.8-62.5] | >0.99 | 19.7  [7.1-27.7] | 13.0  [6.6-72.9] | 0,365 | 12.6  [4.8-31.4] | 15.0  [4.9-46.9] | >0.99 |
| **IL6+ Mon** | 36.6  [5.2-48.6] | 20.8  [11.0-53.8] | >0.99 | 17.9  [5.6-33.7] | 13.7  [6.7-20.2] | >0.99 | 14.8  [3.5-20.7] | 9.1  [6.0-18.5] | >0.99 | 13.1  [6.1-26.3] | 16.1  [8.3-44.2] | >0.99 |
| **CD56 cells** | 316.5  [193.5-609.0] | 254.2  [208.6-556.2] | 0,656 | 353.7  [197.8-611.5] | 391.3  [292.8-547.8] | >0.99 | 271.7  [240.4-537.8] | 243.9  [170.1-426.4] | >0.99 | 355.3  [219.7-526.5] | 294.7  [193.6-454.9] | 0,201 |
| **NKT** | 96.3  [46.2-272.1] | 77.9  [33.5-251.1] | 0,281 | 76.1  [31.9-225.3] | 112.0  [58.7-185.9] | >0.99 | 69.9  [56.0-145.2] | 85.9  [48.20-136.2] | >0.99 | 81.5  [38.9-160.4] | 76.0  [50.1-104.8] | 0,217 |
| **CD56^dim^ NK** | 218.0  [127.7-332.8] | 199.4  [118.0-243.8] | 0,656 | 199.6  [107.7-433.8] | 215.2  [165.0-367.0] | >0.99 | 174.2  [144.6-392.8] | 128.2  [97.3-271.1] | >0.99 | 248.0  [132.0-385.5] | 194.9  [106.8-310.6] | 0,489 |
| **CD56^bright^ NK** | 14.7  [7.2-21.5] | 13.0  [5.1-18.1] | >0.99 | 9.9  [6.2-19.0] | 9.1  [4.1-21.8] | 0.826 | 15.9  [11.7-18.9] | 9.6  [4.8-15.4] | 0,231 | 8.4  [5.7-16.9] | 8.1  [4.3-11.4] | **0.023** |
