# Supplementary material for: Baseline Inflammatory Status Reveals Dichotomic Immune Mechanisms Involved In Primary-Progressive Multiple Sclerosis Pathology
Source: Front Immunol. 2022 Mar 21;13:842354. doi: 10.3389/fimmu.2022.842354 (PMC8977599; doi:10.3389/fimmu.2022.842354)
Supplement: Supplementary file 7 [file Table_3.docx]

**Supplementary Table 3.** Ocrelizumab induced changes in leukocyte blood subset percentages.

Footnote to Supplementary Table 3: Results are shown as Median [25-75% IQR]. Percentages are referred to the subset included into square brackets [ ]. P values were corrected by using Bonferroni test. Gd+/**-**, presence/absence of gadolinium enhancing lesions at baseline; EDA, evidence of disease activity patients at 1 year of follow-up; NEDA, non-evidence of disease activity patients at 1 year of follow-up; 0M, 0 Months (pre-ocrelizumab treatment); 6M, 6 Months of ocrelizumab treatment; *p, corrected p value; IL, interleukin; GM-CSF, Granulocyte-Macrophage Colony-Stimulating Factor; TNFα, Tumor Necrosis Factor-alpha; Treg, regulatory T cells; CM, Central Memory; EM, Effector Memory; TD, Terminally differentiated; IFNγ, Interferon gamma; PD-L1, Programmed Death-ligand 1; Mon, Monocytes; NKT, Natural Killer T cells.

|  | **Gd+ (n=16)** | | | | | | **Gd- (n=53)** | | | | | |
| --- | --- | --- | --- | --- | --- | --- | --- | --- | --- | --- | --- | --- |
| **PERCENTAGES**  **(n=69)** | **EDA (n=6)** | | | **NEDA (n=10)** | | | **EDA (n=12)** | | | **NEDA (n=41)** | | |
|  | **0M** | **6M** | ***p** | **0M** | **6M** | ***p** | **0M** | **6M** | ***p** | **0M** | **6M** | ***p** |
| **CD4 T cells** |  |  |  |  |  |  |  |  |  |  |  |  |
| **Treg [CD4]** | 12.9  [9.0-18.7] | 13.6  [11.0-17.4] | >0.99 | 10.9  [8.6-16.5] | 13.4  [10.0-14.5] | >0.99 | 10.0  [6.7-13.7] | 10.4  [8.9-13.1] | >0.99 | 11.1  [7.4-13.3] | 10.5  [7.8-12.8] | >0.99 |
| **Naïve [CD4]** | 36.2  [25.6-46.9] | 40.4  [33.5-44.4] | >0.99 | 47.8  [30.8-68.3] | 52.2  [37.5-64.5] | >0.99 | 50.9  [46.8-58.7] | 53.6  [45.5-65.3] | >0.99 | 50.8  [35.3-63.0] | 55.3  [47.3-65.5] | **0.004** |
| **CM [CD4]** | 37.9  [27.9-42.1] | 34.6  [32.0-43.0] | >0.99 | 26.9  [15.5-35.9] | 26.2  [20.2-32.7] | >0.99 | 24.4  [17.7-36.1] | 27.8  [20.3-33.5] | >0.99 | 26.8  [21.4-35.0] | 27.4  [20.9-30.9] | 0,301 |
| **EM [CD4]** | 24.6  [15.3-32.0] | 21.4  [14.2-28.0] | >0.99 | 22.0  [6.0-28.1] | 15.6  [9.3-23.9] | >0.99 | 15.1  [7.8-22.2] | 13.1  [8.3-22.1] | >0.99 | 16.6  [9.0-22.3] | 11.8  [8.8-19.3] | **0.040** |
| **TD [CD4]** | 2.6  [1.3-4.5] | 3.3  [1.7-4.0] | >0.99 | 3.2  [2.1-6.6] | 3.2  [1.6-5.2] | >0.99 | 3.3  [1.2-7.6] | 4.0  [1.1-6.2] | >0.99 | 3.2  [1.9-5.2] | 2.6  [1.4-4.0] | **0.002** |
| **IL10+ [CD4]** | 1.2  [0.7-1.7] | 1.1  [0.8-1.7] | >0.99 | 1.1  [0.7-1.9] | 0.9  [0.8-1.1] | >0.99 | 1.3  [0.5-2.2] | 1.2  [0.5-1.5] | >0.99 | 1.0  [0.4-1.5] | 0.8  [0.5-1.5] | >0.99 |
| **GMCSF+ [CD4]** | 7.6  [4.4-8.5] | 7.1  [4.5-8.9] | >0.99 | 9.2  [6.0-14.3] | 9.6  [6.0-10.5] | >0.99 | 7.9  [4.5-11.2] | 7.4  [5.2-9.3] | >0.99 | 6.7  [4.0-9.8] | 7.5  [3.6-10.1] | >0.99 |
| **TNFα+ [CD4]** | 56.3  [38.4-62.3] | 58.9  [38.2-62.9] | >0.99 | 49.6  [26.5-60.8] | 35.5  [26.4-50.4] | 0.334 | 46.6  [35.8-51.6] | 42.1  [35.1-50.0] | >0.99 | 47.9  [31.5-55.7] | 40.6  [31.1-60.1] | >0.99 |
| **IL17+ [CD4]** | 1.1  [0.8-1.5] | 0.9  [0.9-1.0] | >0.99 | 0.9  [0.8-1.5] | 1.2  [0.8-1.8] | >0.99 | 0.9  [0.4-2.4] | 1.3  [0.5-2.1] | >0.99 | 1.0  [0.7-1.4] | 1.0  [0.6-1.6] | >0.99 |
| **IFNγ+ [CD4]** | 12.0  [8.1-16.5] | 10.3  [5.2-15.3] | >0.99 | 9.6  [5.6-15.9] | 8.5  [6.9-11.7] | >0.99 | 9.1  [3.2-11.2] | 6.6  [3.3-8.9] | >0.99 | 7.6  [4.8-10.6] | 6.6  [2.9-9.3] | 0,242 |
| **CD8 T cells** |  |  |  |  |  |  |  |  |  |  |  |  |
| **Naïve [CD8]** | 22.4  [15.5-44.6] | 27.1  [18.0-48.1] | >0.99 | 31.3  [17.0-38.9] | 31.8  [20.1-46.6] | >0.99 | 36.7  [21.0-44.9] | 41.2  [26.7-51.7] | >0.99 | 30.0  [17.7-42.3] | 33.4  [21.2-50.2] | **0.031** |
| **CM [CD8]** | 7.3  [4.6-10.6] | 6.7  [4.6-11.2] | >0.99 | 4.5  [1.8-9.7] | 5.7  [2.4-11.9] | >0.99 | 7.0  [3.3-8.3] | 5.7  [3.2-12.3] | >0.99 | 8.3  [6.5-10.8] | 7.4  [4.6-10.8] | >0.99 |
| **EM [CD8]** | 34.7  [21.9-43.5] | 28.8  [20.0-37.0] | 0.282 | 26.9  [16.9-41.9] | 25.1  [19.3-32.8] | >0.99 | 25.3  [10.6-31.7] | 20.4  [10.8-26.5] | >0.99 | 26.9  [19.3-37.8] | 25.5  [17.3-34.3] | 0,140 |
| **TD [CD8]** | 29.0  [20.2-39.5] | 28.7  [21.6-42.0] | >0.99 | 42.6  [18.4-48.2] | 36.4  [16.4-50.0] | >0.99 | 31.9  [20.4-52.6] | 27.0  [18.9-49.5] | >0.99 | 30.0  [20.8-34.9] | 26.8  [19.7-35.4] | 0,996 |
| **IL10+ [CD8]** | 3.6  [1.3-8.1] | 2.9  [2.5-6.4] | >0.99 | 4.5  [1.8-6.5] | 2.3  [1.5-4.2] | >0.99 | 2.5  [1.0-1.7] | 4.1  [1.7-5.4] | >0.99 | 1.8  [0.7-3.7] | 3.2  [1.4-7.2] | 0,217 |
| **GM-CSF+ [CD8]** | 10.1  [5.5-15.7] | 11.6  [5.5-16.1] | >0.99 | 13.3  [5.1-20.9] | 12.9  [6.1-21.2] | >0.99 | 9.6  [6.1-17.0] | 13.2  [6.2-18.3] | >0.99 | 11.2  [8.0-15.5] | 11.5  [6.5-17.5] | >0.99 |
| **TNFα+ [CD8]** | 38.8  [35.0-57.7] | 45.1  [35.1-56.7] | >0.99 | 57.6  [47.9-67.2] | 55.3  [44.4-67.5] | >0.99 | 44.8  [41.0-54.1] | 41.7  [40.2-45.3] | >0.99 | 51.7  [42.9-65.1] | 47.3  [31.5-58.3] | 0,089 |
| **IL17+ [CD8]** | 3.1  [2.1-4.4] | 2.5  [1.6-4.7] | >0.99 | 2.5  [1.5-4.8] | 2.2  [1.5-5.2] | >0.99 | 2.9  [1.5-4.4] | 3.4  [2.2-7.3] | >0.99 | 2.9  [1.6-4.5] | 3.3  [1.7-6.2] | 0,526 |
| **IFNγ+ [CD8]** | 32.1  [19.2-46.1] | 27.6  [18.3-43.6] | >0.99 | 42.6  [25.0-52.1] | 35.6  [23.7-51.5] | >0.99 | 30.9  [26.8-38.3] | 26.2  [22.9-33.1] | 0.989 | 34.4  [20.5-49.0] | 26.7  [16.0-41.4] | **0.004** |
| **Monocytes** |  |  |  |  |  |  |  |  |  |  |  |  |
| **PD-L1 [Mon]** | 33.3  [12.9-56.8] | 16.4  [11.4-47.9] | >0.99 | 21.8  [15.0-29.8] | 20.8  [15.2-28.6] | >0.99 | 13.4  [5.1-19.7] | 9.8  [7.7-23.3] | >0.99 | 19.4  [8.3-52.9] | 15.0  [7.3-34.0] | 0,193 |
| **IL1B+ [Mon]** | 2.7  [1.8-42.2] | 6.5  [0.9-25.5] | >0.99 | 5.8  [3.3-7.6] | 2.7  [1.7-9.4] | >0.99 | 2.1  [0.9-14.0] | 3.9  [1.7-12.4] | >0.99 | 9.6  [2.4-26.3] | 5.8  [2.1-18.1] | 0,072 |
| **IL10+[Mon]** | 10.7  [4.0-13.0] | 5.7  [3.0-10.5] | >0.99 | 7.7  [2.1-12.0] | 4.6  [2.6-11.9] | >0.99 | 4.7  [2.7-7.2] | 5.0  [2.2-5.6] | >0.99 | 7.9  [3.1-24.9] | 4.9  [2.2-19.1] | 0,462 |
| **TNF+ [Mon]** | 13.2  [4.4-22.1] | 9.7  [3.4-14.0] | >0.99 | 10.6  [4.9-15.0] | 5.0  [3.9-11.4] | 0.387 | 7.2  [3.2-14.2] | 8.7  [4.5-10.8] | >0.99 | 11.9  [3.9-37.1] | 11.3  [3.5-24.2] | 0,262 |
| **IL12+ [Mon]** | 20.2  [6.4-27.9] | 11.7  [4.2-26.9] | >0.99 | 12.9  [8.6-22.7] | 15.3  [12.0-20.1] | >0.99 | 9.5  [6.3-15.8] | 8.6  [6.2-20.8] | >0.99 | 10.3  [4.5-26.0] | 11.8  [3.0-26.6] | >0.99 |
| **IL6+ [Mon]** | 12.7  [6.7-27.6] | 9.0  [5.6-16.8] | >0.99 | 9.4  [7.0-15.5] | 5.2  [4.3-9.9] | 0.387 | 7.1  [4.4-14.2] | 4.9  [3.7-9.0] | >0.99 | 13.5  [5.0-29.8] | 7.9  [4.4-23.7] | 0,728 |
| **CD56 cells** |  |  |  |  |  |  |  |  |  |  |  |  |
| **NKT [CD56]** | 20.6  [9.7-48.2] | 18.8  [10.9-46.0] | >0.99 | 34.8  [10.3-40.1] | 36.7  [19.1-45.3] | >0.99 | 21.0  [8.3-27.9] | 24.1  [10.7-35.5] | >0.99 | 20.5  [5.7-35.4] | 21.0  [7.5-35.5] | >0.99 |
| **CD56^dim^ NK [CD56]** | 60.9  [24.8-79.8] | 65.1  [18.6-79.4] | >0.99 | 63.4  [57.5-78.8] | 61.6  [52.7-78.8] | >0.99 | 67.8  [21.4-72.2] | 59.5  [21.2-73.5] | 0.277 | 59.9  [17.1-75.6] | 60.6  [25.6-72.2] | 0,991 |
| **CD56^bright^ NK [CD56]** | 3.6  [1.4-6.7] | 4.3  [0.5-7.2] | >0.99 | 2.0  [1.5-6.0] | 2.2  [1.3-5.3] | 0.826 | 4.0  [1.8-6.4] | 2.7  [1.6-4.1] | >0.99 | 2.1  [0.7-3.2] | 1.7  [0.7-3.0] | >0.99 |
